# Suicidal ideation, burnout, and their correlation among health care workers at the end of the fourth wave of the COVID-19 pandemic in Alborz Province, Iran

**DOI:** 10.3389/fpsyt.2023.1261105

**Published:** 2023-10-13

**Authors:** Rahim Badrfam, Atefeh Zandifar, Nami Mohammadian Khonsari, Mostafa Qorbani

**Affiliations:** ^1^Department of Psychiatry, Imam Hossein Hospital, Alborz University of Medical Sciences, Karaj, Iran; ^2^Social Determinants of Health Research Center, Alborz University of Medical Sciences, Karaj, Iran; ^3^Student Research Committee, Alborz University of Medical Sciences, Karaj, Iran; ^4^Non-Communicable Diseases Research Center, Alborz University of Medical Sciences, Karaj, Iran; ^5^Chronic Diseases Research Center, Endocrinology and Metabolism Research Institute, Tehran University of Medical Sciences, Tehran, Iran

**Keywords:** COVID-19, suicidal ideation, burnout, health personnel, Iran

## Abstract

**Background:**

During the COVID-19 pandemic, Health Care Workers (HCWs) were more vulnerable than ever to Burnout and Suicidal thoughts due to stressful work conditions. This study, investigated the level of Burnout and Suicidal thoughts among HCWs during the fourth wave of the pandemic in Alborz Province in Iran and compared it with the conditions at the beginning of the pandemic.

**Methods:**

A total of 305 HCWs from 3 referral hospitals for COVID-19, including 155 men and 150 women, participated in the study. A cross-sectional study was carried out with a sample of HCWs dealing with COVID-19 patients using the available sampling method. The results of this online survey, which was conducted from June 7 to July 5, 2021 (at the end of the Fourth Wave of the COVID-19 Pandemic in Iran), have been compared with the conditions of the First Wave of the Pandemic (from February 24 to April 27, 2020). The participants were evaluated by the Beck Scale for Suicidal Ideations (BSSI) and Maslach Burnout Inventory (MBI).

**Results:**

The mean age of the participants was 36.34 ± 7.37. The means of Suicide Index (SI), Emotional Exhaustion (EE), Depersonalization (DP), and Personal Accomplishment (PA) scores were 0.76 ± 1.74, 19.94 ± 4.69, 4.92 ± 1.51, and 31.30 ± 5.88, respectively. SI and PA were significantly higher in workers other than nurses and physicians and EE was higher among workers with night shifts (*p* value<0.05 in all indices). Age had a significant and negative correlation with EE (*p* value<0.01) and DP (p value<0.05) and a significant and positive correlation with PA (*p* value<0.01).

**Conclusion:**

This study showed a high level of SI and Burnout indices among HCWs in the fourth wave of the pandemic in Iran. Paying attention to the factors affecting the development of social capital and creating health policy changes may be effective in reducing Burnout indices and high Suicide index among HCWs.

## Introduction

Higher levels of burnout, depression, and suicide have been reported among Health Care Workers (HCWs) due to stressful work conditions (1). During the COVID-19 pandemic, HCWs were more vulnerable than ever to burnout (2), chronic fatigue (3), post-traumatic stress disorder (PTSD) (4), and suicidal thoughts (5) due to exposure to infection and risk of transmission, staff shortages, lack of personal protective equipment, and aggravated work stress (6).

Various studies have emphasized the development of burnout syndrome among HCWs during the COVID-19 pandemic and based on many studies, suicidal thoughts among HCWs had a high prevalence in that period (7). Also, the relationship between burnout and suicidal thoughts among HCWs is an issue of concern for mental health experts (8). Meanwhile, female HCWs were more vulnerable to burnout, which has been attributed to factors such as less work-life balance (9).

Burnout syndrome is considered a long-term response to chronic emotional and interpersonal stress in the workplace (10). This syndrome is defined as a psychological syndrome with three dimensions, emotional exhaustion (EE), depersonalization (DP), and decreased sense of personal accomplishment (PA) (11).

In a study conducted in Australia, despite the low number of COVID-19 cases, mental HCWs reported significant levels of psychological distress and professional burnout during the pandemic. Related risk factors such as challenging work environment, long working hours, high-intensity work, and regular exposure to discomfort and death of patients have been mentioned as psychological distresses that have an effect on the formation of these symptoms at the professional and patient-related levels (12). The rise in the fear of contracting COVID-19 among HCWs while on duty has resulted in a decline in job satisfaction and an increase in psychological distress. Consequently, this has led to elevated stress and anxiety levels, which in turn have increased the likelihood of HCWs leaving their jobs both within the organization and the profession (13).

Related to this, negative effects on the quality of life and medical services of healthcare workers (HCWs) during the recent pandemic have been reported (14). The severe shortage of HCWs to care for the huge number of patients with COVID-19 and the related medical and nursing care needs, severe limitations of personal care equipment, stigma toward them, being accused of conspiracy and moral harm to them and countless cases of this kind have been associated with reduced quality of life and increased risk of emotional exhaustion among HCWs (15). On the other hand, emotional exhaustion and depersonalization are effective in the formation of psychological distress among HCWs (16) and it is emphasized that work-related burnout may manifest with psychiatric symptoms such as depression, anxiety, and irritability (17).

HCWs are facing unprecedented challenges during the COVID-19 pandemic, which is causing a great deal of uncertainty and unpredictability. This situation is causing work-related stress that can lead to physical exhaustion, emotional distress, and stigmatization. These factors can have a significant negative impact on the mental health of HCWs (18, 19). They face various related problems, one of which is the significant stress and anxiety experienced by different groups of nurses and physicians while caring for patients with COVID-19. This stress has been linked to mental and psychological health problems among them (20, 21).

The COVID-19 pandemic has brought about an increase in suicidal ideations. A systematic review and meta-analysis have identified several risk factors that contribute to this, including low social support, sleep disorders, quarantine, loneliness, and mental health problems. However, among HCWs, high physical and mental exhaustion, as well as poorer self-reported physical health, were identified as the main risk factors (22). A recent meta-analysis highlights a strong correlation between depression and burnout in nursing samples, particularly among those who work in stressful environments and often experience burnout and depression (23). One meta-analysis identified burnout, depression, comorbid mental illness, and stress as the strongest suicide risk factors among medical students (24). On the other hand, according to some reviews, Physician burnout and depression have increased in recent years, while suicide rates have remained relatively the same. Individual factors such as a history of mental health problems and systemic factors such as work compression, and lack of control over one’s professional life have been proposed as factors affecting job burnout and depression (25).

Iran was among the countries that the COVID-19 pandemic entered in a short period after its origin in Wuhan, China. The pandemic brought daily increases in cases and deaths for months. After the end of the fourth wave, the pandemic entered its fifth wave in Iran with the highest numbers of daily new cases and deaths. During the week of June 7th, 2021, there were 59,771 new confirmed cases, which is 11% less than the previous week. Additionally, there were 970 weekly deaths, marking a 19% decrease from the prior week. In the week leading up to July 5th, 2021, there were 83,054 new COVID-19 cases (a 16% increase from the previous week) and 916 deaths (a 6% increase from the previous week) (15).

The effects of working in high-risk conditions related to the spread of the COVID-19 pandemic for HCWs in Iran were associated with high levels of burnout and symptoms of mental health problems such as depression and suicidal thoughts at the beginning of the pandemic (26, 27). HCWs in this study included all types of physicians (general and specialist-including medical interns and residents), all nursing categories (nurses, care assistants, etc.), and all hospital personnel other than physicians and nurses who interact with each other to provide services to patients with COVID-19.

This study was conducted to investigate the level of burnout and suicidal thoughts among HCWs during the fourth wave of the pandemic in Iran and to compare it with the conditions at the beginning of the pandemic along with rooting the causes of their formation in terms of demographic characteristics and working conditions.

## Materials and methods

### Study design

This study was conducted at the end of the fourth wave of the COVID-19 pandemic in the Alborz Province of Iran, as one of the provinces with the highest rate of cases and daily deaths among the 31 provinces of Iran at that time. This was the biggest COVID-19 wave since the spread of the pandemic to Iran, starting immediately after the end of the third wave from March 22 to June 7, 2021. After the forth wave, Iran faced the worst COVID-19 wave during the pandemic which had the highest number of cases and daily deaths (15).

The objective of this study is based on a protocol (28) to conduct cross-sectional and cohort studies to comprehensively assess the mental health of HCWs dealing with COVID-19. The study aims to design both short-term and long-term diagnostic and therapeutic interventions. This study protocol includes cross-sectional studies and interventions at Alborz University of Medical Sciences teaching hospitals. Quantitative, qualitative, and mixed-method studies have been conducted to measure mental health disorders among HCWs, and some results have been published (29, 30).

Iran has a population of 87.92 million people. Alborz province, with 17 cities and 3.212 million people, is densely populated (31). The sample size was determined based on similar studies at all levels. The design of the study was carried out cross-sectionally using the available sampling method and was implemented from June 7 to July 5, 2021. The results of this online survey, have been compared with the conditions of the First Wave of the Pandemic (from February 24 to April 27, 2020).

A number of 305 healthcare workers from a total of three general academic hospitals in Alborz Province of Iran, who were responsible for the diagnosis, treatment, care, and administrative affairs of patients during the peak of the COVID-19 pandemic, were included in the study.

They included women and men with doctorate-level education (M.D. and Ph.D.) and less than doctorate as all types of physicians (general and specialist- including medical interns and residents), all nursing categories (nurses, care assistants, etc.), and hospital personnel other than physicians and nurses, who were working in departments of respiratory emergency, internal medicine, infectious diseases and COVID-19 specific intensive care units (ICU) of these three referral hospitals. Their hiring status at the time of the study was official, contractual, or temporary employment. A group of them were in direct exposure to patients with COVID-19 and a few of them had no direct contact with these patients and worked in the administrative departments of these parts of the hospitals. They worked in the form of three types of fixed daily, fixed night, or cycling shifts. The shifts in these three hospitals were 8 or 12 h in two forms: part-time (eight or fewer shifts per month) and full-time (more than eight shifts per month).

General questions and questionnaires were provided to the participants electronically. The online survey link was shared through WhatsApp and email, in Persian (also known as Farsi), which is the commonly spoken language in Iran. The web-based survey was designed to ensure that each participant could only take the survey once. First, the objectives of the study were explained to the participants and they were assured of the confidentiality of all answers. The participants were assured that they could withdraw from the study at any stage of the survey. At the end of the survey, no participant withdrew from the study. Also, the participants were asked not to share the information related to the questionnaire and the content of the survey with their colleagues (in order to control the social contagion effect). Participation in this study was completely voluntary and anonymous.

### Inclusion and exclusion criteria

All HCWs over the age of 18 who were in one of the official, contractual, or temporary employment situations with Alborz University of Medical Sciences and were working in one of the respiratory emergency, internal medicine, infectious diseases, and intensive care units (ICU) departments of Imam Ali, Imam Hossein, and Kowsar hospitals were considered to participate in the study. They provide COVID-19 services to hospitalized patients in the identified departments, working daily, night, or cycling shifts. They could also be full-time or part-time employees. After sending the request to participate in the study and providing explanations, they could enter the study through the provided link and have informed consent. Failure to complete the requested information after entering the study was considered an exclusion-criteria of the study.

### Data collection

Before entering the desired questionnaires page, first the informed consent section (by expressing a voluntary willingness to participate in the study) was available, and then the demographic information and other personal information including age, gender, education level, number of years of work experience, job category, employment status, Marital status, history of mental illness (by asking the separate question, “Have you been diagnosed with depression, anxiety, or other psychiatric diagnoses before the COVID-19 pandemic?”), direct exposure to a patient with COVID-19, type of work shift (daily, night, cycling) were requested. The status of this group of HCWs who performed their daily activities in these special centers for the care of patients with COVID-19 was clarified in terms of the number of total HCWs in each department per month and the amount of their monthly salary. The information related to standardized and self-reported questionnaires, Beck Scale for Suicidal Ideation (BSSI), and the Maslach burnout questionnaire, were obtained from the participants in the next step. The recorded information based on the mentioned questions and questionnaires was entered in the Statistical Package of Social Sciences (SPSS) software in a categorized form and was available to a biostatistician for final review and statistical analysis.

### Procedure

#### The site

This study was conducted in three general teaching hospitals in Alborz Province, Iran, which were considered referral centers for patients with COVID-19 during the peak of the pandemic. Alborz Province, with a population equal to three million two hundred thousand people, is one of the most populous and immigrant-friendly provinces of Iran and is in the neighborhood of Tehran Province (Iran’s capital) (31), and at the time of the study, during the fourth wave of the COVID-19 pandemic in Iran, it had one of the highest daily infected cases and death rates related to the pandemic (32). Alborz University of Medical Sciences covers all three hospitals where the study was conducted (Imam Hossein, Imam Ali, and Kawsar). At the time of the study, respiratory emergency, internal medicine, infectious diseases, and intensive care unit (ICU) departments were providing services to patients with COVID-19. The total number of inpatient beds related to COVID-19 in these three hospitals was a total of 540 beds in respiratory emergency, internal medicine, and infectious diseases departments and 50 ICU beds specific for COVID-19. The capacity of all these beds was used during the fourth wave of the pandemic and after that in the subsequent waves to admit patients with COVID-19.

#### Selection of participants

An invitation and a link to participate in the study were sent to 400 HCWs who met the inclusion criteria. Out of them, 305 HCWs responded and entered the study, resulting in a response rate of 76.3%. In this study, 50.8% of the participants were male, the mean age of the participants was 36.34 ± 7.37 (range 20–54), and 37% had MD or Ph.D. educational degrees. In addition, 56.4% had a work experience of 5 years and above, 63% were married, and 75.1% had no previous history of mental illnesses. All other basic characteristics are summarized in Table 1.

**Table 1:** Basic characteristics of participants.

Variable		Number	Percentage
Sex	Male	155	50.8
	Female	150	49.2
Education level	MD, Ph.D. (Doctorate)	192	63
	Doctorate >	113	37
Work experience (In years)	5>	133	43.6
	5–10	110	36.1
	>10	62	20.3
Job category	Physicians^1^	110	36.1
	Nursing Categories^2^	126	41.3
	Others^3^	69	22.6
Hiring status	Official	55	18
	Contractual employment	123	40.4
	Temporary	127	41.6
Marriage status	Single	113	37
	Married	192	63
Mental illness History	Yes	76	24.9
	No	229	75.1
Direct exposure	Yes	263	86.2
	No	42	13.8
Shift type	Day	108	35.4
	Night	60	19.7
	Cycling (Day and Night)	137	44.9
Shift number in months	8 ≥	137	44.9
	> 8	168	55.1
Income (in Million Rial*)	≥ 100	139	45.6
	100 >	166	54.4
SI	No	238	78
	Yes	67	22
EE	No	92	30.2
	Yes	213	69.8
DP	No	198	64.9
	Yes	107	35.1
PA	No	161	52.8
	Yes	144	47.2

Suicide Ideation (SI), Emotional Exhaustion (EE), Depersonalization (DP), Personal Accomplishment (PA), Doctor of Medicine (MD), Doctor of Philosophy (Ph.D.) (Academic Doctorate other than MD).

Cutoff values for SI:0) > 0YES), EE: 18 (>18Yes, DP: (>5YES), PA: 40 (40 > Yes).

1 Refers to all types of physicians (general and specialist- including medical interns and residents).

2 Refers to all nursing categories (nurses, care assistants, etc.).

3 Refers to all Hospital personnel other than physicians and nurses.

* Rial is the currency of Iran) an income of 100 million rials per month is considered the average income of an average urban family in terms of economic ability in Tehran, the capital of Iran).

The equivalent value of Rial is 0.000002 US dollars.

Based on the literature review, the following points can be made regarding some variables presented in Table 1.

Three factors related to stress have been proposed regarding the work experience variable: psychological well-being, physical health, and job satisfaction (33). Nurses with less work experience tend to experience burnout in different aspects of their work (34). In relation to hiring status, some studies suggest that temporary workers experience more job burnout and intention to leave than permanent (official) workers (35). In some studies, unmarried nurses had double the burnout rate of married nurses with children (36).

### Measurement scale

#### Beck Scale for Suicidal Ideation (BSSI)

BSSI is an instrument that is widely used to evaluate different aspects of suicide (37). It is a clinical research instrument to quantify and evaluate suicidal intention, which is identified in factor analysis with 3 significant factors of active suicidal desire, specific plans for suicide, and passive suicidal desire (38). This self-assessment tool is designed to measure attitudes, thoughts, and planning for suicide (39) and has 19 items, each item is rated from 0 to 2, and higher scores indicate more intense suicidality (40). Some commentaries introduce the first five items as screening questions (41). BSSI measures having suicide ideation (1–5), preparation for suicide (6–19), and the decision to suicide (20–38). A score of 1 or 2 was considered as indication of the presence of suicidal ideation (42).

The psychometric assessment of BSSI has been carried out in the general population in Iran before COVID-19 Pandemic. In examining the validity, reliability, and factor structure of BSSI in the general population of Tehran, Cronbach’s alpha coefficients in the screening part and the whole scale were reported to be satisfactory (>0.8). The scores of both the screening part and the total scale were higher in people with a history of suicide attempts and had a positive correlation with depression and a negative correlation with social support. In this way, the Persian translation of BSSI was introduced with desirable psychometric properties in the research setting (43). We administered this questionnaire to evaluate suicidal ideation in HCWs who treated COVID-19 patients. The results are presented in the following section.

#### Maslach Burnout Inventory (MBI)

MBI is the most widely used self-administered questionnaire designed to evaluate 3 components of burnout syndrome (44, 45). These 3 components include emotional exhaustion, depersonalization, and reduced personal accomplishment (46). This questionnaire has 22 items which are divided into 3 subscales. The items are scored on a 7-point scale ranging from)0: never (to)6: every day (. The total score of each subscale is calculated by summing its items. Nine items in the Emotional Exhaustion (EE) subscale, 5 items in the Depersonalization (DP) subscale, and 8 items in the Reduced Personal Accomplishment (PA) subscale are the components of this questionnaire. Higher degrees of experienced burnout can be seen in higher degrees of EE and DP and lower degrees of PA (47). Scores higher than 18, 5 in EE and DP and lower than 40 in AP were considered as moderate to severe cases, respectively [(0–18: Low, 19–26: Moderate, 27–54: High) for EE, (0–5: Low, 6–9: Moderate, 10–30: High) for DP and (40–48: Low, 34–39: Moderate, 0–33: High) for PA is for the target] (48).

The validity and Reliability of the Persian version of MBI have been evaluated and confirmed. Three hundred thirty-one employees of factories and public jobs (all forms of legitimate employment) in Iran participated in a study whose aim was instrument (the third version of MBI that used in this study) standardization. According to the results of this study (49), the item total correlation and internal consistency (total alpha) were 0.79, 0.85, and 0.87, respectively, the intraclass correlation coefficient was 0.87, indicating good test–retest reliability (r = 0.87, *p* < 0.01), and the construct validity of the scale using exploratory factor analysis, showed 3 factors with eigenvalues greater than 1 (1 and 5 items: α = 0.72; 2, 4 items: α = 0.78; 3, 6 statements: α = 0.69).

### Statistical analysis

Data were analyzed using Statistical Package of Social Sciences (SPSS) software version 24(SPSS Inc., Chicago, IL, United States). The Kolmogorov–Smirnov test was used to assess the normal distribution of the variables. Categorical variables were presented as “frequency (N=) and percentage (%) and continuous variables as mean and standard deviation (SD). The Chi-square test and Fisher’s exact test were used to compare qualitative data. Due to the non-normal distribution of continuous variables, Spearman’s correlation coefficient correlation was used to analyze the correlation between total score of continuous variables, namely, occupational exhaustion (EE), depersonalization (DP), reduced Personal Accomplishment (PA), Suicidal Ideation (SI), and age. Univariate and multivariate logistic regression analyses were used to determine the association of demographic variables and EE, DP, PA, and SI. A two-tailed *p*-value<0.05 was considered significant.

### Ethical considerations

The ethics committee of Alborz University of Medical Sciences approved the proposal for this research project on 29/05/2021 (IR.ABZUMS.REC.1400.068). The participants were informed about the research objectives and how to participate before entering the study. They were assured that all information provided by them would be confidential and will not be shared with any individual or group. The participants completed and sent the informed consent form before entering to the study. In this study, all components of the Declaration of Helsinki and its appendix were considered.

## Results

Regarding the outcomes of this study, means and ranges were 0.76 ± 1.74 (range 0–11) for SI, 19.94 ± 4.69 (range 10–34) for OR, 4.92 ± 1.51 (range 2–9) for DP, and 31.30 ± 5.88 (range 20–43) for PA.

Table 2 shows the findings of categorical SI and Maslach Burnout Inventory subscales (EE, PA, DP) across demographic variables. SI and DP were significantly higher among workers other than nurses and physicians, whereas EE and PA were significantly higher among nursing categories (based on Chi-square or Fisher’s exact tests as relevant).

**Table 2:** Comparison of suicide ideation (SI) and Maslach burnout inventory subscales categories across demographic variables.

Variable		SI		*p*-value	EE		*p*-value	DP		*p*-value	PA		*p*-value
		No	Yes		No	Yes		No	Yes		No	Yes	
Sex	Male	84 (52.2)	71 (49.3)	0.64 (Fisher)	53 (57.6)	102 (47.9)	0.13 (Fisher)	83 (52.2)	71 (49.3)	0.64 (Fisher)	41 (25.5)	35 (24.3)	0.89 (Fisher)
	Female	77 (47.8)	73 (50.7)		39 (42.4)	111 (52.1)		76 (47.8)	73 (50.7)		120 (74.5)	109 (75.7)	
Education level	MD, Ph.D. >	96 (59.6)	96 (66.7)	0.23 (Fisher)	61 (66.3)	131 (61.5)	0.44 (Fisher)	128 (64.6)	64 (59.8)	0.45 (Fisher)	96 (60.4)	96 (66.7)	0.28 (Fisher)
	≥ MD, Ph.D.	65 (40.4)	48 (33.3)		31 (33.7)	82 (38.5)		70 (35.4)	43 (40.2)		63 (39.6)	48 (33.3)	
Work experience (in years)	5>	77 (47.8)	56 (38.9)	0.11	31 (33.7)	102 (47.9)	0.04*	81 (40.9)	52 (48.6)	0.12	77 (48.4)	56 (38.9)	0.09
	5-10	58 (36.0)	52 (36.1)		36 (39.1)	74 (34.7)		70 (35.4)	40 (37.4)		57 (35.8)	52 (36.1)	
	>10	26 (16.1)	36 (25.0)		25 (27.2)	37 (17.4)		47 (23.7)	15 (14)		25 (15.7)	36 (25)	
Job category	Physicians^1^	64 (39.8)	46 (31.9)	0.001>*	30 (32.6)	80 (37.6)	0.001>*	69 (34.8)	41 (38.3)	0.02*	62 (39.0)	46 (31.9)	0.001>*
	Nursing Categories^2^	75 (46.6)	51 (35.4)		22 (23.9)	104 (48.8)		75 (37.9)	51 (47.7)		75 (47.2)	51 (35.4)	
	Others^3^	22 (13.7)	47 (32.6)		40 (43.5)	29 (13.6)		54 (27.3)	15 (14.0)		22 (13.8)	47 (32.6)	
Hiring status	Official	26 (16.1)	29 (20.1)	0.6	26 (28.3)	29 (13.6)	0.006*	39 (19.7)	16 (15)	0.007*	25 (15.7)	29 (20.1)	0.06
	Contractual Employment	75 (46.6)	48 (33.3)		29 (31.5)	94 (44.1)		67 (33.8)	56 (52.3)		74 (46.5)	48 (33.3)	
	Temporary	60 (37.3)	67 (46.5)		37 (40.2)	90 (42.3)		92 (46.5)	35 (32.7)		60 (37.7)	67 (46.5)	
Marriage status	Single	71 (44.1)	42 (29.2)	0.009* (Fisher)	32 (34.8)	81 (38.0)	0.60 (Fisher)	73 (36.9)	40 (37.4)	1.00 (Fisher)	71 (44.7)	42 (29.2)	0.006* (Fisher)
	Married	90 (55.9)	102 (70.8)		60 (65.2)	132 (62.0)		125 (63.1)	67 (107)		88 (55.3)	102 (70.8)	
Mental illness History	Yes	41 (25.5)	35 (24.3)	0.89 (fisher)	24 (26.1)	52 (24.4)	0.77 (fisher)	50 (25.3)	26 (24.3)	0.89 (fisher)	41 (25.8)	35 (24.3)	0.79 (Fisher)
	No	120 (74.5)	109 (75.7)		68 (73.9)	161 (75.6)		148 (74.7)	81 (75.7)		118 (74.2)	109 (75.7)	
Direct exposure	Yes	146 (90.7)	117 (81.3)	0.01* (fisher)	67 (72.8)	196 (92.0)	0.001>* (fisher)	166 (83.8)	97 (90.7)	0.11 (Fisher)	144 (90.6)	117 (81.3)	0.02* (Fisher)
	No	15 (9.3)	27 (18.8)		25 (27.2)	17 (8.0)		32 (16.2)	10 (9.3)		15 (9.4)	27 (18.8)	
Shift type	Day	46 (28.6)	62 (43.1)	0.02*	51 (55.4)	57 (26.8)	0.001>*	75 (37.9)	33 (30.8)	0.42	45 (28.3)	62 (43.1)	0.01*
	Night	38 (23.6)	22 (15.3)		8 (8.7)	52 (24.4)		36 (18.2)	24 (22.4)		38 (23.9)	22 (15.3)	
	Cycling (Day and Night)	77 (47.8)	60 (41.7)		33 (35.9)	104 (48.8)		87 (43.9)	50 (46.7)		76 (47.8)	60 (41.7)	
Number of shifts (in months)	8 ≥	63 (39.1)	74 (51.4)	0.03* (Fisher)	56 (60.9)	81 (38.0)	0.001>* (Fisher)	88 (44.4)	49 (45.8)	0.90 (Fisher)	62 (39.0)	74 (51.4)	0.03* (Fisher)
	> 8	98 (60.9)	70 (48.6)		36 (39.1)	132 (62.0)		110 (55.6)	58 (54.2)		97 (61.0)	70 (48.6)	
Income (in million)	≥ 10	74 (46.0)	65 (45.1)	0.90 (Fisher)	48 (52.2)	91 (42.7)	0.13 (Fisher)	92 (46.5)	47 (43.9)	0.71 (fisher)	72 (45.3)	65 (45.1)	1 (Fisher)
	> 10	87 (54.0)	79 (54.9)		44 (47.8)	122 (57.3)		106 (53.5)	60 (56.1)		87 (54.7)	79 (54.9)	

Suicide Ideation (SI), Emotional Exhaustion (EE), Depersonalization (DP), Personal Accomplishment (PA), Doctor of Medicine (MD), Doctor of Philosophy (Ph.D.) (Academic Doctorates other than MD).

The data are presented as frequency (percentage) (*n* (%)).

Cutoff values for SI: 0) > 0YES), EE: 18 [>18Yes, DP: (>5YES), PA: 40 (40 > Yes)].

*p-values under 0.05 are considered statistically significant (based on Chi-square or Fisher’s exact tests as relevant).

1 Refers to all types of physicians (general and specialist).

2 Refers to all nursing categories (nurses, care assistants, etc.).

3 Refers to all Hospital personnel other than physicians and nurses.

SI was significantly higher among married people, without direct exposure, with daily shift work and the number of shifts equal to or less than 8 days per month. EE was significantly higher among those with less than five years of work experience, contract workers, those with direct exposure, and those with higher shift numbers and night shifts. DP was significantly higher among contract workers than among official and temporary workers. PA was significantly higher in the married workers, the group without direct exposure, workers with daily shifts, and the number of shifts less than or equal to eight days per month.”

The Spearman’s correlation coefficient results are presented in Table 3. As can be seen, age had a significant and negative correlation with EE and DP and a significant and positive correlation with PA. Moreover, SI had a significant and positive correlation with EE, and EE also had a significant positive correlation with DP and a negative correlation with PA.

**Table 3:** Spearman’s correlation coefficient among Age, SI, and Maslach burnout inventory subscales.

	Age	Overall SI score	Overall EE score	Overall DP score	Overall PA score
Age	1	(−)	(−)	(−)	(−)
Overall SI score	0.001	1	(−)	(−)	(−)
Overall EE score	−0.177**	0.115*	1	(−)	(−)
Overall DP score	−0.142*	0.098	0.372**	1	(−)
Overall PA score	0.166**	−0.110	−0.648**	−0.240**	1

Suicide Ideation (SI), Emotional Exhaustion (OE), Depersonalization (DP), Personal Accomplishment (PA).

*p*-values under 0.05 were considered statistically significant.

* *p*-value <0.05,

** *p*-value <0.01.

SI, EE, and PA were significantly associated with workers other than nurses and physicians and EE also had a significant relationship with working shifts at night (7 pm to 7 am in 12-h shifts and 11 pm to 7 am in 8-h shifts).

Based on this model, DP had a statistically significant relationship with work experience of more than 10 years. These findings are summarized in Table 4.

**Table 4:** The association of demographic and SI, OE, DP, and PA based on univariate and multivariate logistic regression analyses.

Variable		SI		EE		DP		PA	
		Crude	Adjusted	Crude	Adjusted	Crude	Adjusted	Crude	Adjusted
Age		1.03 (1.00–1.06)*	1.02 (0.96–1.08)	0.94 (0.91–0.98)*	0.93 (0.87–1.00)	0.98 (0.95–1.01)	(–)	1.03 (1.00–1.07)*	1.01 (0.96–1.07)
Sex	Male	1	1	1	1	1	1	1	1
	Female	1.12 (0.71–1.75)	(–)	1.47 (0.90–2.42)	1.51 (0.86–2.64)	1.28 (0.80–2.06)	(–)	1.12 (0.71–1.76)	(–)
Education level	MD, Ph.D. (Doctorate)	1	1	1	1	1	1	1	1
	Doctorate >	0.73 (0.46–1.17)	(–)	1.23 (0.73–2.05)	(–)	1.22 (0.75–1.99)	(–)	0.76 (0.47–1.21)	(–)
Work experience (in years)	5>	1	1	1	1	1	1	1	1
	5–10	1.23 (0.74–2.05)	0.99 (0.49–1.99)	0.62 (0.35–1.10)	0.83 (0.35–1.92)	0.89 (0.52–1.50)	0.85 (0.49–1.45)	1.25 (0.75–2.08)	1.03 (0.51–2.07)
	>10	1.90 (1.03–3.50)*	1.10 (0.40–3.03)	0.45 (0.23–0.85)*	1.36 (0.41–4.48)	0.49 (0.25–0.98)*	0.49 (0.24–0.99)*	1.98 (1.07–3.66)*	1.17 (0.42–3.22)
Job category	Physicians^1^	1	1	1	1	1	1	1	1
	Nursing categories^2^	0.94 (0.56–1.59)	1.60 (0.84–3.03)	1.73 (0.95–3.30)	0.61 (0.25–1.44)	1.14 (0.67–1.93)	0.99 (0.57–1.71)	0.91 (0.54–1.54)	1.54 (0.81–2.93)
	Others^3^	2.97 (1.57–5.59)*	3.76 (1.72–8.21)*	0.27 (0.14–0.51)*	0.19 (0.07–0.47)*	0.46 (0.23–0.93)*	0.46 (0.21–1.03)	2.87 (1.52–5.42)*	3.63 (1.66–7.93)*
Hiring status	Official/Permanent	1	1	1	1	1	1	1	1
	Contractual employment	0.57 (0.302–1.09)	(–)	2.90 (1.48–5.69)*	1.73 (0.75–3.98)	2.03 (1.03–4.02)*	(–)	0.55 (0.29–1.06)	(–)
	Temporary	1.00 (0.53–1.88)	(–)	2.18 (1.13–4.19)*	1.01 (0.41–2.45)	0.92 (0.46–1.86)	(–)	0.96 (0.50–1.82)	(–)
Marriage status	Single	1	1	1	1	1	1	1	1
	Married	1.91 (1.19–3.08)*	1.64 (0.94–2.85)	0.86 (0.52–1.44)	(–)	0.97 (0.60–1.59)	(–)	1.95 (1.21–3.15)*	1.67 (0.96–2.91)
Mental illness History	Yes	1	1	1	1	1	1	1	1
	No	1.06 (0.63–1.79)	(–)	1.09 (0.62–1.91)	(–)	1.05 (0.61–1.81)	(–)	1.08 (0.64–1.82)	(–)
Direct exposure	Yes	1	1	1	1	1	1	1	1
	No	2.24 (1.14–4.41)*	0.82 (0.32–2.09)	0.23 (0.11–0.45)*	0.84 (0.29–2.39)	0.53 (0.25–1.13)	0.84 (0.34–2.05)	2.21 (1.12–4.35)*	0.82 (0.32–2.09)
Shift type	Day	1	1	1	1	1	1	1	1
	Night	0.43 (0.22–0.82)*	0.48 (0.21–1.07)	5.81 (2.52–13.40)*	4.84 (1.72–13.65)*	1.51 (0.78–2.92)	(–)	0.42 (0.21–0.80)*	0.48 (0.21–1.07)
	Cycling (Day and Night)	0.57 (0.34–0.96)*	0.60 (0.30–1.18)	2.82 (1.63–4.85)*	2.44 (1.11–5.36)*	1.30 (0.76–2.23)	(–)	0.57 (0.34–0.95)*	0.60 (0.30–1.19)
Number of shifts (in months)	8 ≥	1	1	1	1	1	1	1	1
	> 8	0.60 (0.38–0.95)*	0.82 (0.46–1.46)	2.53 (1.53–4.18)*	1.28 (0.66–2.49)	0.94 (0.59–1.51)	(–)	0.60 (0.38–0.95)*	0.82 (0.46–1.46)
Income (in millions)	≥ 10	1	1	1	1	1	1	1	1
	10 >	1.03 (0.65–1.62)	(–)	1.46 (0.89–2.39)	1.16 (0.58–2.32)	1.10 (0.69–1.77)	(–)	1.00 (0.63–1.58)	(–)

Suicide Ideation (SI), Emotional Exhaustion (EE), Depersonalization (DP), Reduced Personal Accomplishment (PA), Doctor of Medicine (MD), Doctor of Philosophy (Ph.D.) (Academic Doctorates other than MD).

The data are presented as odds ratio (OR) and Confidence Interval (CI) (OR (CI)).

*p-values under 0.05 are considered statistically significant.

Multivariate logistic regression analyses were done for variables (uncategorized) with a P-value of 0.2 ≥ in the crude analysis.

1 Refers to all types of physicians (general and specialist).

2 Refers to all nursing categories (nurses, care assistants, etc.).

3 Refers to all Hospital personnel other than physicians and nurses.

## Discussion

This study examined suicidal ideation and worker burnout among job categories that provide services to patients with COVID-19 at the end of the fourth wave of the pandemic in Iran based on demographic characteristics, work history, and mental illness history. Half of the study participants were men and more than half of them had more than 5 years of work experience. Three-quarters of them also had no history of mental illnesses. SI and PA were significantly higher in workers in the job category other than nurses and physicians, which was also significant in the multivariate logistic regression analysis model. Despite the positive relationship between EE and DP with the nursing categories, no statistically significant relationship between them was obtained based on this regression model. Also, EE was higher among workers with night shifts, which was also found to be significant in the multivariate logistic regression analysis.

In the statistical analysis using Chi-square and Fisher’s exact tests in this study at the end of the fourth wave, work experience of fewer than 5 years, a job in the nursing category, having direct exposure to COVID-19, night shift, and the number of shifts more than 8 days per month was associated with higher levels of EE. Also, jobs in nursing categories and contractual employment conditions were associated with higher levels of DP. A job other than the physicians and nursing categories, being married, not having direct exposure to COVID-19, working daily shifts, and the number of shifts equal to or less than 8 days per month were also associated with higher levels of PA.

In a study that was conducted during the second wave of the COVID-19 epidemic (27 August through 23 October 2020) (50) among Frontline HCWs in Australia, 10.5% of participants in that study had thoughts of suicide or self-harm over a two-week period (7). A higher rate of burnout was also reported among this group of HCWs. In the current study, in multivariable models, a significant relationship was found between having these thoughts and variables such as younger age (⩽30 years *cf.* >50 years;), male gender, increased income worries, and prior mental illness.

The presence of suicidal thoughts in this study that took place in Iran, was 22% among HCWs and in line with the results of the mentioned study, it had a statistically significant relationship with some components of burnout syndrome (EE). One of the reasons for the higher rate of suicidal thoughts in this study may be related to the possible more exposure to environmental risk factors mentioned above (exposure to infection and risk of transmission (51, 52), staff shortages (53), lack of personal protective equipment (54), and aggravated work stress (6, 55)) among the participants of this study. The number of weekly confirmed cases during this one-month survey was between 59,000 and 114,000 (in the whole of Iran) and the time of this study was at the end of the fourth wave and immediately at the beginning of the fifth wave. Meanwhile, in Australia’s second wave, there were 660 to 91 weekly confirmed cases at the time of the study (which appears to be largely independent of population differences in the two countries).

Another important point, comparing these two studies, is the difference in risk factors related to suicidal thoughts (both of which were evaluated based on multivariable statistical models and among a large number of possible risk factors). In this study in Iran, none of the significant variables in the mentioned study in Australia were found to increase the risk of suicidal thoughts (mainly demographic factors) and only having a job other than physicians and nursing categories was the only independent risk factor increasing the risk of suicidal thoughts. Another important point is that in this study, the lowest PA scores were also obtained in the same group of non-physician and non-nurse participants. This was while no direct correlation was found between SI and PA. In this way, in this study, despite SI and PA scores being higher in the non-physician-non-nurse group, no direct relationship was found between the two. It seems that it is necessary to pay attention to other models related to suicide and other non-demographic components.

Our satisfaction in work environments and family life is largely dependent on our interpersonal relationships and friendships (56). Along with such a trend, collective action is also considered as communicative in nature and in the mode of interpersonal interaction and the mode of engagement that shapes interaction (57). Networks of strong interpersonal relationships that develop over time and create a foundation for trust, cooperation, and collective action can be synthesized as “social capital” which is an important variable in conducting health-related research and/or in synthesizing the results from health-related investigations. It may be possible to consider the results of this study in line with the theories presented by Durkheim in the field of suicide and its relationship with social capital (58–61).

According to this, Durkheim saw lower rates of suicide in societies with the highest levels of integration and the highest rates of suicide in societies with a loosening of social bonds (59). Social capital has been the focus of experts in three dimensions: structural, cognitive, and relational, and in practice, it includes complex interrelationships between these three dimensions. The structural aspect shows the existence of a network of access to people and resources, while its other aspects reflect the ability to exchange resources (62). The concept of social capital has been widely reflected in various socio-cultural groups in recent years (63–65). In this way, taking into account the scope of the concept and its practical application in explaining behavioral characteristics has been of interest to experts in the social fields (66). Some have also considered the close relationship between social and cultural capital that is supported by processes of social stratification processes. In this sense, the strengthening of social equality in exchange for the distribution of such capital will be visible at the community level (67). In a multicenter prospective cohort study, social capital among physicians, registered nurses, and assistant nurses was strongly related to job satisfaction and active engagement with clinical improvements (68). In another study of healthcare workers, managers who support collaboration and social interaction among work teams may reduce burnout by promoting social capital (69).

Considering the components of social capital in the form of social associations and networks, norms of reciprocity, and trust (70), it can be expected that in this study, heavy and risky therapeutic activity in high-stress conditions among the group of physicians and nurses has strengthened social cohesion among the group of colleagues (71). This point may have had a protective effect regarding suicidal thoughts in these groups compared to other employees who were not on the front line of dealing with COVID-19.

This is while the overall high suicide index, in its place, clearly raises the difficulty of the conditions and the influence of inappropriate environmental factors in the formation of these thoughts (16). As in a report from Iran about the high prevalence of suicidal thoughts among physicians during the COVID-19 pandemic, other different factors such as the low monthly salaries of physicians especially clinical residents, the requirement to be present in the desired cities of health decision-making systems for new graduates without taking into account any preferences of physicians and non-standard working hours considered as other difficulties they face, especially during the COVID-19 pandemic (72).

On the other hand, the significantly higher level of suicidal thoughts among HCWs in this study compared to the study conducted in Australia, in addition to the need to pay attention to the difference in the tools used, may be related to the influence of factors other than lower social capital in the formation of suicidal thoughts among individuals and social groups. For example, the limitation of opportunities and resources for social and economic recovery from negative consequences of the COVID-19 pandemic, even in the case of high social capital and social cohesion, may be associated with serious harm to mental health, especially among those with the highest exposure to pandemic-related hardships (73). This is the basis for the formation of a point of view in conflict with Durkheim’s theories, in which it is emphasized that not paying attention to the effect of political change as a determining factor for the health of the population and simply aiming for social cohesion can be associated with expected negative consequences in health areas (58).

Such a situation in this study, considering the relative acceptability of social capital in Iranians based on national studies and social analyzes conducted in this field (74, 75), may be significant and referable. The poor economic-social situation in Iran during recent years and its more obvious manifestation during the COVID-19 pandemic in the form of a shortage of personal protective equipment, limitation of human resources, lack of medical equipment for necessary procedures for patients (intensified during the pandemic), inefficient management in controlling the situation, disproportionate distribution of financial and human resources and further, excessive inefficiency in providing vaccination against COVID-19 (76–80) may be the effective factors in increasing the problems related to mental health, including high levels of depression, anxiety, burnout, and suicidal thoughts in HCWs (81, 82).

This inter-relationship can be supported by the relative decrease in the suicide rate in in the United States during the influenza epidemic between 1918–19 simultaneously with the improvement of the economic situation and low unemployment rate, and the increase in the suicide rate during the 1921 pandemic, simultaneously with the economic recession and the increase in the unemployment rate. This was while at the beginning, the increase in social cohesion following the collective misfortunes related to the First World War and the invasion that happened in the first pandemic was mentioned as one of the effective factors in reducing the suicide rate (73).

In the study, which was conducted during the first wave of the COVID-19 pandemic in all provinces of Iran (including Alborz Province, which was the place of this study), in order to investigate occupational burnout syndrome, using the MBI questionnaire among HCWs (April 6 to May 30, 2020), 34.2, 48.7 and 56.1 percent of the participants showed high levels of EE, DP, and PA, respectively (83). These rates in this study at the end of the fourth wave of COVID-19 and among occupational groups similar to the mentioned study, were 69.8, 35.1 and 47.2%, respectively.

In the mentioned study during the first wave, factors such as being in the age range of 20 to 30 years, being female, not having children, having a bachelor’s degree, and working in isolation departments were associated with higher levels of occupational burnout (analysis based on Chi-square test). Also, having a history of physical diseases and psychiatric disorders was suggested as the best predictor of occupational burnout.

As mentioned, among these demographic and environmental factors in this study at the end of forth wave, in multivariate logistic regression analyses, the only significant relationship was obtained with higher levels of EE and working night shifts and higher levels of PA with a job other than the physicians and nursing categories. Also, in this study, having a history of psychiatric disorders had no significant relationship with higher levels of any of the burnout syndrome components. Specifically, the number of HCWs with higher levels of EE in this study increased more than twice in the fourth wave of COVID-19 compared to the first wave in Iran. This situation has been accompanied by a slight decrease in the number of HCWs with higher levels of DP and PA. Regarding the difference created in the EE situation in the interval between the first and fourth waves in Iran, considering the relative difference of the conditions in two different studies (including the study mentioned in the first wave and this study), other groups of health care workers and administrators can benefit from the key findings gained in the study reported herein.

In a similar study in the United States that was conducted for three years, EE was evaluated among HCWs in three time periods before the COVID-19 pandemic (2019) and twice in 2020 and 2021–2022 (12). They proposed exhaustion score clustering in work settings related to the social contagion effect of exhaustion. The results indicated a decrease in the first year among physicians and sharp increases in 2021 in the second year among them and an annual increase in EE in nurses and other groups of HCWs. They indicated an overall increase in EE from 31.8% in 2019 to 40.4% in 2022.

This rate in this study in Iran among all HCWs participating in the study in 2021 and during the peak of the pandemic was 69.8%, which was higher than the rate among HCWs in the United States. This difference may be due to the higher baseline level of EE among HCWs in Iran or its exacerbation due to the addition of problems related to the COVID-19 pandemic such as heavy workload and improper distribution of financial and human resources on top of other common problems mentioned in the previous sections. Also, the amount of EE in this study in Iran was higher in Nursing Categories compared to other groups of HCWs, although this difference was not significant in multivariate logistic regression analyses.

At the same time, considering the lower percentage of HCWs with high levels of DP and RP in this study compared to the first-wave study in Iran and the doubling of HCWs with high EE, it is possible to pay attention to the social contagion effect of exhaustion which was noticed by researchers in the study conducted in the United States. In this regard, social contagion is defined according to the American Psychological Association (APA) Dictionary of Psychology as “the spread of behaviors, attitudes, and affect through crowds and other types of social aggregates from one member to another” (84). Based on the available evidence, such a process can be considered a complex interaction between individual, relational, and social factors (85). Also, social contagion can be related to some behavioral disorders and psychiatric symptoms such as suicide (86) and violence (87).

## Conclusion

This study showed a high level of SI and burnout indices among HCWs in the fourth wave of the pandemic in Iran. Some demographic factors or working conditions seemed effective in the formation and aggravation of this situation. Among the demographic factors, age was the most related to changes in burnout components.

The increase in the number of HCWs with higher levels of EE compared to similar studies in previous waves of the COVID-19 pandemic in Iran may also be interpreted by the social contagion effect of exhaustion. Paying attention to the factors affecting the development of social capital and creating health policy changes as a determining factor of population health may be effective in reducing burnout indices and suicide index among HCWs and considering the limitations related to the studies conducted in this field, can be taken into the attention of experts for future research. Such secondary changes may be achieved through health policymakers’ strengthening of ongoing financial and occupational support for HCWs. This study also provides background information on the present investigation, which will be useful for researchers in other regions and countries, who wish to examine health care institutions and improve working conditions for their employees and even volunteers. Designing and conducting studies in this field by considering the control group in the time before or a significant period after the pandemic, in the current conditions, or in similar pandemics in the future, can be taken into consideration by experts in future research.”

## Limitations

In this study, there was no control group. Therefore, the interpretations made from the statistical results can only be in the form of hypotheses related to the pandemic. Also, the nature of this study is cross-sectional, which prevents the formation of a causal relationship. One limitation of this study is the methodology used, which involved administering online questionnaires and providing explanatory materials in the form of a questionnaire package. This approach was necessitated by the high-risk pandemic situation at the time of the study.

The study was specifically conducted to evaluate the mental health of HCWs in the general hospitals of Alborz province in Iran during the COVID-19 pandemic. Therefore, it is important to note that the results cannot be applied to the overall population of the country. Considering the sample sizes used in similar studies in the Alborz province of Iran, we tried to make this limitation of sampling at the level of the province as minimal as possible.

Although participants were asked not to share survey information to prevent the social contagion effect related to burnout, lack of control remained if sharing occurred. This point is another limitation of the recent study. These considerations can be considered for future research.

## Data availability statement

The raw data supporting the conclusions of this article will be made available by the authors, without undue reservation.

## Ethics statement

The studies involving humans were approved by the Ethics Committee of Alborz University of Medical Sciences – 29/05/2021 (IR.ABZUMS.REC.1400.068). The studies were conducted in accordance with the local legislation and institutional requirements. The participants provided their written informed consent to participate in this study.

## Author contributions

RB: Conceptualization, Investigation, Methodology, Project administration, Resources, Supervision, Validation, Visualization, Writing Original draft. AZ: Conceptualization, Funding acquisition, Investigation, Methodology, Project administration, Resources, Supervision, Validation, Visualization, Writing – review & editing. NM: Conceptualization, Data curation, Investigation, Methodology, Software, Resources, Visualization, Writing – review & editing. MQ: Conceptualization, Data curation, Formal analysis, Methodology, Project administration, Resources, Software, Supervision, Validation, Visualization, Writing – review & editing.
